# Targeted Recruitment of Cross-Kingdom Phosphate-Solubilizing Microbes Drives Asymmetric Rhizosphere Responses Between *Solanum rostratum* and *Cenchrus pauciflorus* Benth. in Sandy Habitats

**DOI:** 10.3390/plants15121837

**Published:** 2026-06-14

**Authors:** Song Yang, Zhen Niu, Yilang Miao, Yujie Chen, Guangchao Lyu, Wenjing Ma, Yang Wang, Linyou Lyu, Xun Tian

**Affiliations:** 1 College of Life Science and Food, Inner Mongolia Minzu University, Tongliao 028000, China; nmdyangsong@163.com (S.Y.); mawj0902@163.com (W.M.); 2 Management Committee of Zhangwu Grassland Ecological Restoration Demonstration Area, Fuxin 123000, China; wangyang491@163.com; 3 Liaoning Institute of Sandy Land Control and Utilization, Fuxin 123000, China; llyou515@126.com

**Keywords:** plant co-invasion, cross-kingdom microbiome, phosphorus-solubilizing microbes, *Solanum rostratum*, *Cenchrus pauciflorus*

## Abstract

In resource-poor sandy habitats, alien plant co-invasion often triggers intense belowground competition mediated by rhizosphere microorganisms. However, the mechanisms by which these plants overcome nutrient limitations remain unclear. Here, we conducted an eight-month in situ monitoring of single- and co-invasion plots of *Solanum rostratum* and *Cenchrus pauciflorus* Benth. in the Horqin Sandy Land. By integrating soil enzyme assays with 16S rRNA and internal transcribed spacer (ITS) amplicon sequencing, we characterized their rhizosphere microbial community assembly. Co-invasion exposed both species to convergent biotic stress, characterized by the significant enrichment of the pathogenic fungi *Didymella* and *Pseudogymnoascus* (linear discriminant analysis (LDA) > 4.0). To mitigate these pressures, the dominant competitor, *S. rostratum*, specifically recruited a cross-kingdom phosphate-solubilizing consortium comprising *Bacillus* and *Penicillium* (LDA > 4.0). This targeted recruitment significantly enhanced rhizosphere activities, increasing phosphatase and sucrase to 86.10 U/g and 2.17 U/g, respectively, thereby maintaining available phosphorus at a high level (35.55 mg/kg). Conversely, the subordinate competitor, *C. pauciflorus*, lost key native stress-resistant bacteria such as *Rubrobacter* (relative abundance dropping from 5.39% to 3.27%) and failed to recruit effective microbes, leading to the rapid depletion of available phosphorus (dropping to 21.38 mg/kg). Ultimately, under dual nutrient and pathogenic stress, the precise recruitment and functional integration of cross-kingdom phosphate-solubilizing microbes are strongly linked to the divergent belowground competitive outcomes between these co-invading plants.

## 1. Introduction

The global invasion of alien plants increasingly poses a severe threat to native biodiversity and ecosystem functioning [1]. In arid and semi-arid sandy ecosystems (e.g., the Horqin Sandy Land in China), extreme soil impoverishment renders nutrient limitation the primary abiotic bottleneck restricting plant growth and community assembly [2]. Given the low soil organic matter content and the predominance of insoluble phosphorus (P) in sandy soils, the drastic reduction in P availability leaves plants in a chronic state of severe “P limitation” [3]. Recently, accumulating evidence has shown that multiple alien plant species frequently exhibit “co-invasion” within the same habitat. This high degree of spatiotemporal niche overlap intensely exacerbates belowground resource competition [4,5].

Furthermore, high-density plant co-invasion not only accelerates the depletion of available soil nutrients but also alters the rhizosphere microenvironment, inducing the enrichment and cross-species transmission of soil-borne pathogenic fungi—a phenomenon known as the “pathogen spillover” effect [6,7]. This pathogen sharing and habitat degradation induced by co-invasion force plants to simultaneously confront the dual pressures of extreme P deficiency and pathogen stress during belowground competition [8]. Under such multiple environmental stresses, traditional competition theories emphasizing phenotypic plasticity or physical resource preemption fail to fully elucidate how certain alien plants establish asymmetric competitive advantages, particularly regarding the underlying belowground driving mechanisms [9].

Increasing evidence indicates that the rhizosphere microbiome, functioning as the plant’s “second genome,” plays a pivotal role in assisting hosts to withstand complex biotic and abiotic stresses [10]. While early microecological studies were primarily confined to the response and succession of single bacterial communities, recent ecological research highlights that “cross-kingdom microbial networks” comprising both bacteria and fungi are more critical for maintaining rhizosphere ecosystem multifunctionality and enhancing plant adaptability [11,12,13]. In particular, in extremely P-deficient environments, phosphate-solubilizing microbes (PSMs) with potent nutrient-mobilizing capabilities, such as specific *Bacillus* and *Penicillium* taxa, can efficiently lyse insoluble phosphates by synergistically secreting extracellular phosphatases and organic acids, thereby overcoming habitat P limitation [14]. Competitively dominant plants can proactively increase the input of root exudates (e.g., saccharide carbon sources) to establish a “carbon-for-phosphorus” nutrient acquisition strategy in the rhizosphere, thereby precisely inducing and maintaining this cross-kingdom phosphate-solubilizing functional consortium [15].

As a typical agro-pastoral ecotone and ecologically fragile region in northern China, the Horqin Sandy Land has faced severe pressure from land degradation and retrograde vegetation succession in recent years [2,16]. In this extremely oligotrophic habitat characterized by intense aeolian sand activities, the soil structure is loose and severely depleted of organic matter. The vast majority of P exists in highly recalcitrant inorganic mineral forms, resulting in an extreme scarcity of available P supply. From an ecophysiological perspective, P is not only a core element driving deep root penetration and extension but also the material basis for maintaining plant energy metabolism (e.g., adenosine triphosphate (ATP) synthesis) and crucial stress tolerance, such as drought and disease resistance. Therefore, amid the exceptionally intense multi-species belowground competition in the Horqin Sandy Land, the capacity to efficiently mobilize and utilize insoluble soil P has become a decisive factor dictating whether alien invasive plants can overcome habitat bottlenecks, suppress competitors, and ultimately dominate community succession [3,17].

This study focuses on the co-invasion system of typical invasive plants in the Horqin Sandy Land: *Solanum rostratum* and *Cenchrus pauciflorus* Benth. (also known as *Cenchrus spinifex*). Relying on continuous in situ sampling monitoring throughout the plant growing season, we integrated amplicon (16S rRNA and ITS) high-throughput sequencing with soil eco-enzymology to systematically dissect the cross-kingdom assembly patterns and carbon–phosphorus metabolic mobilization dynamics of the rhizosphere microbiomes of both species under single- and co-invasion models. Based on this, we propose the following hypothesis: Under the shared pathogen stress induced by co-invasion, the dominant invasive species can establish an asymmetric rhizosphere competitive advantage by actively recruiting cross-kingdom phosphate-solubilizing microbial consortia to overcome sandy land P limitation [18]. Furthermore, this study aims to reveal the rhizosphere microecological regulatory mechanisms driving the asymmetric outcomes of alien plant co-invasion in oligotrophic sandy habitats. This will provide novel perspectives to enrich the theories of asymmetric competition and cross-kingdom microecological assembly in extreme habitats, offering scientific support for formulating precision management strategies against multi-species synergistic invasions in fragile northern sandy lands based on microbiome manipulation. In this study, the ‘asymmetric competitive advantage’ within the belowground ecosystem is explicitly operationalized and characterized by three core rhizosphere indicators: (1) the differential accumulation of available nutrients, particularly available phosphorus (AP); (2) the divergent activities of key metabolic enzymes, such as sucrase (SUC) and phosphatase (PHO); and (3) the asymmetric assembly of functional microbial communities, specifically the targeted recruitment of phosphate-solubilizing consortia versus the loss of native stress-resistant bacteria.

## 2. Results

### 2.1. Co-Invasion Triggers Asymmetric Responses in Soil Carbon and Phosphorus Metabolism

**Figure 1:**
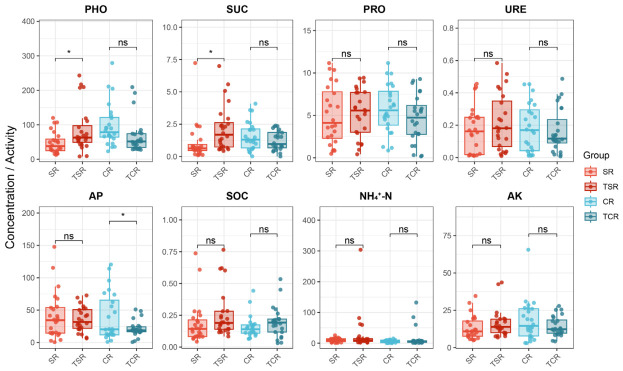
Differential responses of physicochemical properties and enzyme activities in the rhizosphere of *S. rostratum* and *C. pauciflorus* under mono- and co-invasion. Box-and-jitter plots show the variations in eight core soil parameters across four rhizosphere groups. SR: *S. rostratum* mono-invasion; TSR: *S. rostratum* co-invasion; CR: *C. pauciflorus* mono-invasion; TCR: *C. pauciflorus* co-invasion. Asterisks (*) indicate significant differences (*p* < 0.05), while “ns” indicates no significance (*p* > 0.05), based on one-way analysis of variance (ANOVA) followed by Tukey’s HSD test (*p* < 0.05). Abbreviations: PHO, phosphatase; SUC, sucrase; URE, urease; PRO, protease; AP, available phosphorus; SOC, soil organic carbon; AK, available potassium; NH_4_^+^-N, ammonium nitrogen.

Crucially, analysis of the bulk soil controls across the three geographic sampling sites revealed no significant differences in basic physicochemical properties or enzyme activities (Supplementary Table S1). This confirms that the observed divergent metabolic responses in the rhizosphere are strictly driven by the specific plant invasion models, effectively ruling out potential site-specific background effects. Measurements of core rhizosphere physicochemical properties and enzyme activities revealed that *Solanum rostratum* and *Cenchrus pauciflorus* exhibited significantly divergent metabolic response trajectories under co-invasion (Figure 1). Regarding metabolic mobilization, the rhizosphere phosphatase (PHO) and sucrase (SUC) activities of *S. rostratum* in the co-invasion group (TSR) were significantly upregulated compared to the single-invasion group (SR) (*p* < 0.05). Previous studies indicate that the synergistic upregulation of enzymes related to carbon and phosphorus cycling is a crucial microecological mechanism by which plants respond to nutrient limitation [19]. Furthermore, although the available phosphorus (AP) concentration in the co-invasion rhizosphere of *S. rostratum* (TSR) was numerically lower (35.55 mg/kg) than its single-invasion counterpart (SR, 40.9 mg/kg), this difference was not statistically significant (*p* > 0.05). Although available potassium (AK) content showed no significant differences across treatments, the increased activities of C and P metabolic enzymes provided a fundamental basis for resource acquisition by *S. rostratum* in this oligotrophic environment.

In contrast to the response pattern of *S. rostratum*, *C. pauciflorus* under co-invasion (TCR) failed to exhibit a similar compensatory increase in enzyme activities. Its rhizosphere PHO activity showed a decreasing trend (though not statistically significant compared to the single-invasion CR group), and the rhizosphere available phosphorus (AP) content was significantly depleted relative to the CR group (*p* < 0.05). In severely P-limited sandy habitats, the significant depletion of soil AP not only indicates the degradation of the effective nutrient supply system in the plant rhizosphere but also physiochemically corroborates the subordinate, suppressed status of *C. pauciflorus* within the co-invasion dynamic [6,20].

### 2.2. Shared Stress and Specific Recruitment: Identification of Cross-Kingdom Core Microbial Biomarkers

**Figure 2:**
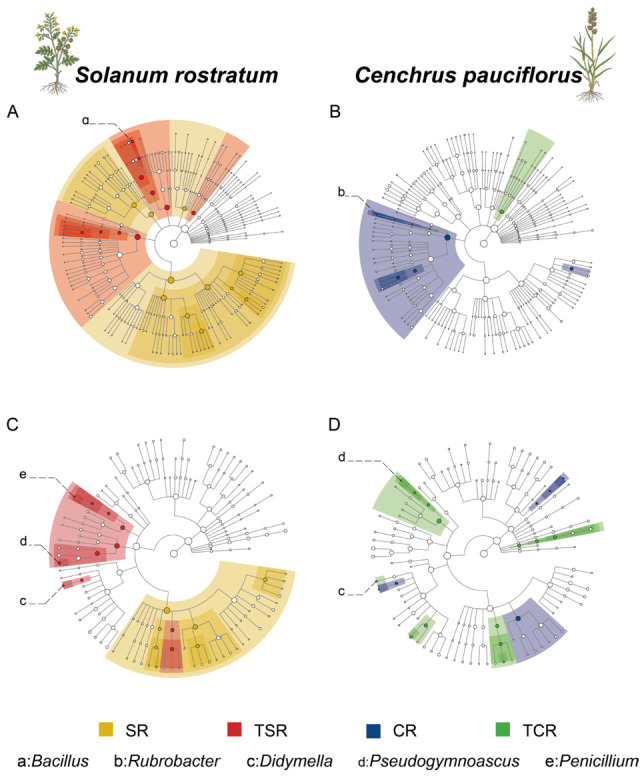
Identification and targeted recruitment of cross-kingdom core microbial biomarkers under shared pathogenic stress. LEfSe cladograms representing the phylogenetic distribution of bacterial and fungal lineages enriched in different invasion treatments. The panels display the taxonomic succession of bacterial (**A**) and fungal (**C**) communities in the *S. rostratum* rhizosphere, alongside bacterial (**B**) and fungal (**D**) communities in the *C. pauciflorus* rhizosphere. Circles radiating from the center represent taxonomic levels from phylum to genus. Node size is proportional to the average relative abundance of the taxon. Colored nodes indicate taxa significantly enriched in corresponding groups: SR (*S. rostratum* mono-invasion, yellow), TSR (*S. rostratum* co-invasion, red), CR (*C. pauciflorus* mono-invasion, dark blue), and TCR (*C. pauciflorus* co-invasion, green). Five key cross-kingdom genera driving the asymmetric rhizosphere response are highlighted: a: *Bacillus* and e: *Penicillium* (cross-kingdom phosphorus-solubilizing community specifically enriched in TSR); c: *Didymella* and d: *Pseudogymnoascus* (shared pathogenic/stress-indicator fungi convergently enriched in TSR and TCR); and b: *Rubrobacter* (dominant indigenous bacterium in CR, which was significantly depleted in TCR).

To elucidate the underlying drivers of the aforementioned asymmetric rhizosphere metabolic responses, we utilized Linear Discriminant Analysis Effect Size (LEfSe) with a logarithmic LDA score threshold of 4.0 to identify cross-kingdom microbial biomarkers (Figure 2). Data analysis revealed a response pattern characterized by the coexistence of convergent stress and specific community assembly. Regarding shared environmental pressures, the rhizosphere microbiomes of both the TSR and TCR groups exhibited significant enrichment of *Didymella* and *Pseudogymnoascus*. *Didymella* comprises various soil-borne plant pathogenic fungi, and its enrichment indicates a pathogen spillover effect induced by high-density spatiotemporal plant overlap [21]. Conversely, the convergent enrichment of *Pseudogymnoascus* reflects a community-level stress response to extreme abiotic conditions, such as severe cold, drought, and oligotrophy [22].

Regarding specific community assembly, *S. rostratum* demonstrated a capacity for the targeted integration of specific plant growth-promoting microbes (PGPMs). The TSR group was specifically enriched with functional taxa, notably *Penicillium* and *Bacillus*. Within the microecological network of *C. pauciflorus*, the stress-resistant indicator taxon *Rubrobacter*, which was dominant during single invasion (CR group), experienced significant depletion under co-invasion pressure (TCR group). *Rubrobacter* exhibits robust tolerance to extreme desiccation and barren habitats; thus, the precipitous decline in its relative abundance directly compromised the basal stress resistance of the *C. pauciflorus* rhizosphere system [23].

### 2.3. Cross-Kingdom Phosphate-Solubilizing Guilds Drive Rhizosphere Phosphorus Mobilization

**Figure 3:**
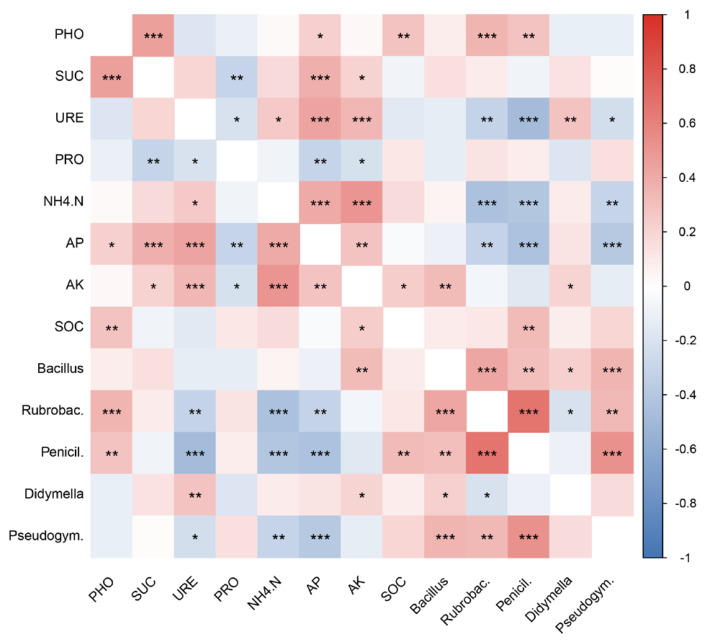
Spearman rank correlation heatmap between cross-kingdom core microbial functional groups and soil carbon/phosphorus metabolism. The heatmap illustrates Spearman’s rank correlation coefficients between the relative abundances of five key microbial genera (identified via LEfSe) and eight physicochemical/enzymatic parameters. Red squares indicate positive correlations, while blue squares indicate negative correlations. Color intensity reflects the magnitude of the correlation coefficient (r). Asterisks denote statistical significance: * *p* < 0.05, ** *p* < 0.01, *** *p* < 0.001. Due to formatting constraints, certain microbial genus names are abbreviated in the figure (*Pseudogym.* for *Pseudogymnoascus*; *Rubrobac.* for *Rubrobacter*; *Penicil.* for *Penicillium*). Highly significant positive correlations (e.g., between *Rubrobacter*/*Penicillium* and PHO) highlight that the colonization of targeted cross-kingdom microbes directly drives inorganic phosphorus mobilization in the sandy microenvironment.

Spearman rank correlation analysis between the identified microbial biomarkers and physicochemical parameters further elucidated the driving pathways of rhizosphere nutrient mobilization (Figure 3). Heatmap analysis revealed a significant positive correlation between the relative abundance of the fungal genus *Penicillium* and PHO activity, suggesting that the enrichment of this functional taxon is a key biological pathway for alleviating host P limitation [24]. Notably, *Penicillium* abundance exhibited a highly significant negative correlation with residual soil AP and NH_4_^+^-N contents. Ecological mechanism studies indicate that highly efficient phosphate-solubilizing microbes (e.g., certain *Penicillium* strains) not only secrete extracellular enzymes to mineralize organic P but also robustly stimulate host root proliferation, thereby driving the “predatory” uptake of available nutrients, such as P and N, by the plant [25]. In extremely P-deficient sandy habitats, the mobilized available P is instantaneously absorbed and assimilated by the massive plant “nutrient sink,” leading to rapid nutrient turnover and strong biological immobilization. This spatiotemporal dynamic between mobilization and uptake plausibly explains the apparent negative correlation between the abundance of growth-promoting consortia and the free soil available nutrient pool. Furthermore, although *Bacillus* abundance was not directly correlated with PHO activity, it was significantly and positively correlated with AK content, reflecting its synergistic role as a multifunctional plant growth-promoting bacterium (PGPB) in mobilizing complementary nutrients such as potassium.

Within the *C. pauciflorus* rhizosphere microbiome, the abundance of the native stress-resistant taxon *Rubrobacter* exhibited a highly significant positive correlation with PHO activity. The significant depletion of this taxon in the TCR group directly eliminated the fundamental functional source maintaining PHO activity. This loss prevented the upregulation of phosphatase expression under stress conditions, ultimately triggering a substantial collapse of the AP supply system. Additionally, correlation network analysis revealed that the general upregulation of SUC activity lacked significant positive correlations with any of the aforementioned microbial biomarkers. This lack of correlation suggests that the increase in sucrase activity likely stems directly from host root exudate carbon inputs rather than from the secondary metabolites of these specific microbes [26]. This decoupling between metabolic enzyme activity and microbial abundance indicates an adaptive basal carbon allocation strategy employed by the plant to proactively nourish the rhizosphere ecosystem. Based on these correlation patterns, we postulate that the synergistic interaction between the colonization of specific functional consortia and plant rhizosphere carbon allocation constitutes a key mechanism driving the asymmetric mobilization of soil nutrients under co-invasion.

### 2.4. Overall Assembly Characteristics of the Rhizosphere Microbiome

**Figure 4:**
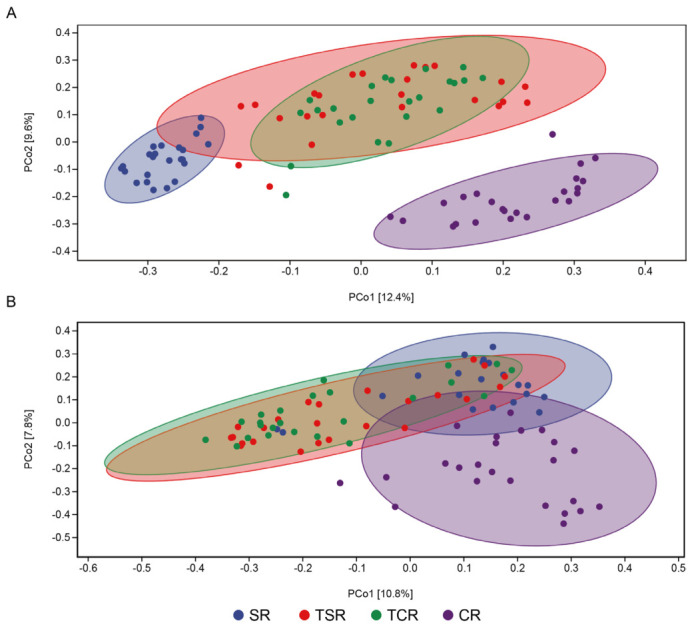
Principal Coordinate Analysis (PCoA) illustrating the beta diversity succession of rhizosphere microbial communities under mono- and co-invasion. PCoA plots based on Bray–Curtis dissimilarities depict the structural variations in bacterial (**A**) and fungal (**B**) communities across the four experimental groups. Different colors represent the four experimental groups, and the corresponding colored shaded areas (ellipses) represent the 95% confidence intervals for each group. Significant structural differences were statistically validated using PERMANOVA. Abbreviations: SR, *Solanum rostratum* mono-invasion; TSR, *S. rostratum* co-invasion; CR, *Cenchrus pauciflorus* mono-invasion; TCR, *C. pauciflorus* co-invasion.

To elucidate the impact of invasion modes on the overall architecture of the rhizosphere microbiome, we evaluated the beta diversity succession patterns of bacterial and fungal communities across groups based on Principal Coordinate Analysis (PCoA) (Figure 4). Data analysis revealed that under co-invasion stress, bacterial and fungal communities exhibited significantly asynchronous convergent response patterns. Under single-invasion conditions, the rhizosphere bacterial and fungal communities of both *Solanum rostratum* (SR group) and *Cenchrus pauciflorus* (CR group) displayed high host specificity, showing significant separation in the ordination space (PERMANOVA, bacteria: pseudo-*F* = 12.35, df = 1; fungi: pseudo-*F* = 6.30, df = 1; both *p* = 0.001). The occurrence of co-invasion disrupted these original assembly trajectories. The spatial distribution patterns indicated that the bacterial community structures of the co-invasion groups (TSR and TCR) completely deviated from their respective single-invasion baselines (pseudo-*F* = 7.18 and 5.48, respectively, df = 1, *p* = 0.001) and highly overlapped within the ordination space. Statistical testing confirmed no significant difference in the overall bacterial community structure between the TSR and TCR groups (pseudo-*F* = 1.19, df = 1, *p* = 0.142). This strong convergence of the overall bacterial community suggests that under extreme nutrient competition and shared pathogen stress, environmental filtering overrides host genetic control, forcing distinct plant species to construct a homogenized basal microecological chassis [27].

In contrast, the fungal community response to co-invasion exhibited a higher degree of host dependency. Although the TSR and TCR groups also showed a clear clustering trend in the fungal PCoA plot, significant structural differences were maintained between them (pseudo-*F* = 1.62, df = 1, *p* = 0.035). This asynchrony in cross-kingdom responses reflects the differential sensitivities of distinct microbial kingdoms to habitat stress: bacteria, owing to their rapid turnover rates, are more prone to convergent succession driven by environmental factors such as resource scarcity; conversely, fungi, due to their deeper physical and physiological binding with host roots, retain partial host specificity even under extreme competitive pressure [28].

At higher taxonomic levels, the bacterial communities across all groups were consistently dominated by Actinobacteriota and Proteobacteria, while the fungal communities maintained an absolute predominance of Ascomycota (Figure S1). These high-abundance phyla play a keystone role in maintaining the basal metabolism and structural stability of the ecosystem in arid sandy lands [29]. Against the backdrop of macro-community convergence (bacteria) or partial convergence (fungi), the succession of the specific functional taxa identified earlier (Section 3.2 and Section 3.3) emerges as the key driver of the competitive landscape. The ecological process by which the dominant species (*S. rostratum*) establishes its competitive advantage relies not on a drastic remodeling of the overall microecological chassis, but rather on the targeted recruitment and fine-scale regulation of specific cross-kingdom phosphate-solubilizing functional modules (e.g., *Bacillus* and *Penicillium*) within a highly convergent community framework.

## 3. Discussion

### 3.1. Cross-Kingdom Convergent Succession and Asymmetric Rhizosphere Competition Driven by Co-Invasion

This study demonstrates that in oligotrophic sandy habitats, the directional succession and reorganization of specific rhizosphere microbial functional modules constitute the core mechanism driving the asymmetric outcomes of *Solanum rostratum* and *Cenchrus pauciflorus* co-invasion. The dual habitat pressures of intense interspecific competition and extreme barrenness compel the overall rhizosphere microbial community structures of both competitors to undergo significant convergent succession, accompanied by the spillover of shared pathogenic fungi [27,30]. However, the establishment of the competitive dominance of the dominant species (*S. rostratum*) is not predicated on the systemic remodeling of the overall community taxonomic architecture. Instead, against the backdrop of overall community convergence, this plant selectively overcomes the phosphorus limitation bottleneck of sandy habitats by precisely recruiting cross-kingdom nutrient-mobilizing consortia (*Bacillus* and *Penicillium*). In contrast, under co-invasion pressure, the subordinate species (*C. pauciflorus*) not only fails to construct an effective compensatory growth-promoting network but also loses its original core stress-resistant microecological taxon (*Rubrobacter*). Consequently, it falls into a vulnerable state characterized by a dual decline in nutrient acquisition and environmental tolerance. This response pattern provides a microecological dimension to traditional plant niche and resource competition models [31,32,33], suggesting that under extreme environmental stress, plant regulation of specific cross-kingdom functional modules may serve as a key driver shaping competitive dynamics.

### 3.2. Environmental Filtering and Asynchrony in Cross-Kingdom Community Responses

The mechanisms by which extreme environmental pressures shape plant rhizosphere microbial community assembly remain a core topic in microecology [34,35]. We observed that co-invasion not only enriched shared soil-borne pathogenic indicator taxa such as *Didymella* [4] but also drove the rhizosphere bacterial communities of *S. rostratum* and *C. pauciflorus* to significantly deviate from their original trajectories under single invasion, exhibiting highly homogenized characteristics. This response pattern diverges significantly from the traditional view frequently emphasized in this field, which posits that alien invasive plants possess strong host-specific filtering capabilities [36]. This phenomenon suggests that under the dual stress of extreme oligotrophy and high-density interspecific competition in the Horqin Sandy Land, the driving role of environmental filtering in bacterial community assembly significantly overrides the specific selection effects of the host plants [27]. Notably, cross-kingdom microbial responses exhibit distinct asynchrony during this successional process: unlike the highly convergent bacterial communities, fungal communities retained significant host-specific structures. We postulate that this difference in cross-kingdom assembly likely originates from distinct micro-niche-dependent strategies. Bacteria, with shorter generation times, are more susceptible to the drastically fluctuating physicochemical environment of the rhizosphere. In contrast, fungi, owing to the deeper physical and physiological anchoring between their complex hyphal networks and host roots, exhibit stronger ecological inertia under extreme stress [28].

### 3.3. Carbon–Phosphorus Metabolic Decoupling and Targeted Recruitment of Cross-Kingdom Growth-Promoting Consortia

Against the backdrop of overall rhizosphere microbiome convergence, the targeted regulation of specific cross-kingdom functional modules by the dominant species becomes critical for establishing its competitive advantage. This study confirms that *S. rostratum* specifically integrated a collaborative growth-promoting consortium comprising *Bacillus* and *Penicillium*, which was accompanied by a significant upregulation of rhizosphere sucrase (SUC) activity. Traditional perspectives frequently attribute the synergistic upregulation of soil carbon and phosphorus cycling-related enzyme activities to a general expansion of total microbial biomass [27]. However, the lack of a significant correlation between enhanced SUC activity and the abundance of targeted recruited genera in our study challenges the traditional explanatory model based on synchronous biomass growth. This decoupling between metabolic enzyme activity and microbial abundance suggests that the surge in SUC activity might reflect a potential “proactive carbon investment” strategy by the host plant. Specifically, the dominant plant may increase the targeted input of specific root exudates (e.g., saccharides or organic acids) as a regulatory mechanism to induce and sustain the colonization of specific cross-kingdom phosphate-solubilizing consortia [37,38].

In this cross-kingdom synergistic system, the enrichment of *Penicillium* significantly drives the enzymatic mineralization of organic P (the PHO pathway). Meanwhile, *Bacillus*, which has no direct association with PHO, may participate in non-enzymatic P solubilization by mobilizing complementary nutrients (e.g., AK) or acting as “helper bacteria” to secrete low-molecular-weight organic acids, thereby exerting a cross-kingdom synergistic enhancement effect [25,39]. This targeted assembly of complementary functions provides a fundamental basis for *S. rostratum* to achieve asymmetric competitive dominance in nutrient-limited habitats.

### 3.4. Underlying Mechanisms of Rapid Nutrient Turnover and Defensive Collapse

Interestingly, despite the significant enrichment of the cross-kingdom phosphate-solubilizing consortium in the TSR group, the rhizosphere AP concentration did not increase but rather maintained a level (35.55 mg/kg) with no significant difference from the SR group (40.9 mg/kg). This phenomenon can be explained by the intense belowground competitive dynamics. Under co-invasion stress, the dominant plant exhibits a dramatically heightened nutrient demand to sustain its competitive growth, leading to the rapid root uptake of the newly mobilized AP [20]. Concurrently, the highly active recruited microbial community also immobilizes a portion of the phosphorus for its own proliferation [40]. Consequently, the targeted recruitment of phosphate-solubilizing microbes effectively compensates for the massive phosphorus consumption, preventing the depletion of the soil AP pool and maintaining it at a stable level comparable to single-invasion conditions.

A key finding of this study is the highly significant negative correlation between the enrichment of cross-kingdom phosphate-solubilizing functional modules and soil free available phosphorus (AP) content. In conventional agricultural or greenhouse simulation systems, a high abundance of phosphate-solubilizing microbes typically correlates positively with the soil’s available P accumulation [14]. However, in extremely nutrient-poor sandy habitats, this negative correlation highlights an intense dynamic of “rapid nutrient turnover and plant immobilization.” We postulate that the free P mobilized by microbes cannot accumulate long-term in the soil matrix; rather, it is rapidly absorbed and assimilated by the root system of the competitively dominant plant, which acts as a robust nutrient sink [40,41]. This highly coupled “mobilization–uptake” process within the rhizosphere microdomain not only explains the apparent negative correlation between growth-promoting microbe abundance and available nutrients but also confirms the exceptionally high resource acquisition and conversion efficiency of *S. rostratum* during belowground microdomain competition [18,42].

**Figure 5:**
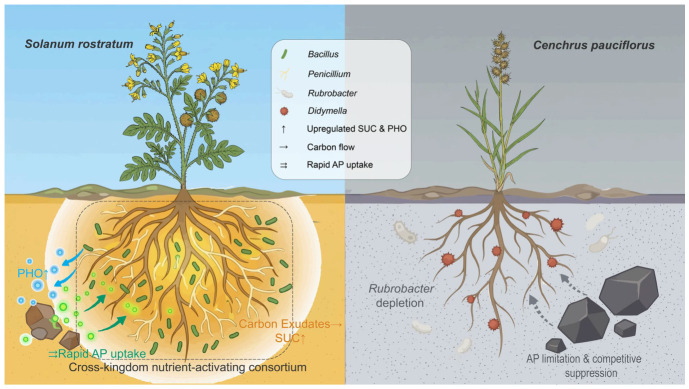
Conceptual model of the cross-kingdom microecological mechanisms driving the asymmetric co-invasion of *Solanum rostratum* and *Cenchrus pauciflorus* in a nutrient-poor sandy habitat. The left panel illustrates the targeted breakthrough strategy of the dominant species, *S. rostratum*. By actively investing carbon exudates (coupled with upregulated SUC activity), the plant specifically recruits a cross-kingdom nutrient-activating consortium composed of *Bacillus* and *Penicillium*. This microbial guild significantly upregulates phosphatase (PHO) activity to mobilize insoluble phosphorus (rocks), and the newly released available phosphorus (AP) is rapidly taken up by the root system (Rapid AP uptake), securing a competitive advantage. The right panel depicts the defensive collapse of the subordinate species, *C. pauciflorus*. Under dense spatial competition and shared infection by the pathogenic fungus *Didymella*, its indigenous stress-resistant bacterium (*Rubrobacter*) undergoes severe depletion. The dismantling of this basal microecological defense impairs baseline nutrient mobilization, ultimately driving the plant into AP limitation and competitive suppression. The base graphical elements of this conceptual model were generated with the assistance of artificial intelligence tools, followed by extensive manual scientific annotation and editing by the authors.

In stark contrast, the competitive decline of *C. pauciflorus* does not merely stem from the compression of its physical survival space. The significant depletion of its native core stress-resistant microecological taxa (e.g., *Rubrobacter*) under convergent successional pressure directly compromised the microecological functions required to maintain basal phosphatase activity. This functional loss further exacerbated the P-limited state of the species, constituting a crucial intrinsic mechanism for its competitive disadvantage (the driving mechanisms of cross-kingdom microecological interactions and asymmetric competition are summarized in Figure 5).

### 3.5. Limitations, Theoretical Significance, and Future Perspectives

Although this study systematically elucidates the belowground microecological regulatory network driving the outcomes of plant co-invasion, the research design entails certain limitations. First, our inference of the active carbon allocation process in plants was primarily based on extracellular enzyme activity (EEA) phenotypes, lacking quantitative profiling of the in situ dynamic chemical composition of root exudates. Future studies urgently need to incorporate spatial metabolomics and stable isotope probing (e.g., ^13^C-DNA SIP) to establish a direct causal chain of evidence for carbon–phosphorus material fluxes [43]. Second, the co-occurrence networks derived from high-throughput sequencing primarily reveal systemic correlations; the absolute causal contribution of this cross-kingdom network to plant competitive ability requires further validation through synthetic community (SynCom) inoculation experiments [44,45].

Nevertheless, by utilizing in situ data from natural extreme habitats, this study provides a novel theoretical perspective for invasion ecology. The findings suggest that the competitive mechanisms of invasive plants in oligotrophic habitats extend beyond mere physical competition for space and nutrients, profoundly relying on the targeted recruitment and cross-kingdom regulation of specific microecological functional modules. The discovery of this mechanism holds significant practical implications for the management of alien plants in fragile northern ecosystems: For such synergistic invasion systems, future comprehensive management strategies should not only focus on the physical or chemical eradication of the plants themselves but also explore ecological control technologies based on targeting microecological networks (e.g., precisely disrupting the *Bacillus*-*Penicillium* cross-kingdom consortium). By attenuating the microecologically targeted empowerment of dominant invasive species, it is promising to constrain their invasive potential at the level of rhizosphere interactions [46,47].

## 4. Materials and Methods

### 4.1. Site Description and Sample Collection

The Horqin Sandy Land in Inner Mongolia was selected as the study area. To evaluate the ecological effects of plant co-invasion, three standard 30 m × 30 m large plots were established: Sihetun in Horqin District (co-invasion of *Solanum rostratum* and *Cenchrus pauciflorus* with similar abundances; 43°40′12.5″ N, 122°7′35.8″ E), Heilongba in Kailu County (dominant single-invasion population of *S. rostratum*; 43°27′23.9″ N, 120°47′33.0″ E), and Dongfeng Forestry Farm in Kailu County (dominant single-invasion population of *C. pauciflorus*; 43°28′19.7″ N, 121°41′18.8″ E). Within each large plot, three independent 3 m × 3 m quadrats were randomly established as biological replicates. The sampling period spanned the entire plant growing season (April to October), with regular sampling conducted on the 20th of each month. An additional sampling was performed in August (August 5 and 20) during the rapid plant growth phase, totaling eight sampling time points.

Rhizosphere soil samples were collected using a sterile elution and centrifugation method. After excavating intact plant roots, loose bulk soil was gently shaken off. Approximately 15 g of roots with tightly adhering soil was excised and placed into sterile centrifuge tubes. The roots were thoroughly washed with a 0.86% sterile NaCl solution and then removed. The resulting eluate was centrifuged at 4000× *g* for 30 min at 24 °C. After discarding the supernatant, the soil pellet at the bottom was collected as the rhizosphere soil sample, snap-frozen in liquid nitrogen, and stored in a −80 °C ultra-low temperature freezer for subsequent nucleic acid extraction and high-throughput sequencing [48,49]. The remaining air-dried roots and sieved soil from bare patches within the quadrats (bulk soil control) were utilized to determine soil enzyme activities and physicochemical properties, respectively.

### 4.2. Determination of Soil Physicochemical Properties and Enzyme Activities

Basic soil physicochemical properties were quantitatively analyzed using standard colorimetric and titration methods. Specifically, soil organic carbon (SOC) content was determined using the potassium dichromate–sulfuric acid oxidation method; available phosphorus (AP) was measured via the molybdenum–antimony anti-spectrophotometric method; ammonium nitrogen (NH_4_^+^-N) and available potassium (AK) were extracted and quantitatively determined using a standardized soil nutrient analysis system coupled with specific rapid-test chemical reagents [50].

Extracellular enzyme activities in the rhizosphere soil characterize the turnover rates of carbon, nitrogen, and phosphorus within the habitat. Soil sucrase (SUC) activity was determined using the 3,5-dinitrosalicylic acid colorimetric method; urease (URE) activity was measured by the sodium phenate–sodium hypochlorite colorimetric method; protease (PRO) activity was quantified using the ninhydrin colorimetric method; and phosphatase (PHO) activity was quantified at a specific wavelength using the disodium phenyl phosphate colorimetric method [2,51].

### 4.3. Genomic DNA Extraction and Amplicon Sequencing

Total genomic DNA was extracted from the rhizosphere soil pellets using the MagBeads FastDNA Kit for Soil (MP Biomedicals, Santa Ana, CA, USA) strictly following the manufacturer’s standard protocols. DNA concentration and purity were assessed using a NanoDrop NC2000 spectrophotometer (Thermo Fisher Scientific, Waltham, MA, USA) and 0.8% agarose gel electrophoresis. For bacterial communities, the V3-V4 hypervariable region of the 16S rRNA gene was PCR-amplified using the specific primers 338F (5′-ACTCCTACGGGAGGCAGCA-3′) and 806R (5′-GGACTACHVGGGTWTCTAAT-3′) [52]. For fungal communities, the ITS1 region was amplified using the specific primers ITS5 (5′-GGAAGTAAAAGTCGTAACAAGG-3′) and ITS2 (5′-GCTGCGTTCTTCATCGATGC-3′) [53]. PCR products were purified using Vazyme VAHTS DNA Clean Beads (Vazyme Biotech Co., Ltd., Nanjing, China) and quantified with Quant-iT PicoGreen (Thermo Fisher Scientific, Waltham, MA, USA) before being pooled in equimolar amounts to construct the sequencing libraries. Finally, paired-end high-throughput sequencing (2 × 250 bp) was performed on the Illumina NovaSeq 6000 (Illumina Inc., San Diego, CA, USA) platform by Shanghai Personal Biotechnology Co., Ltd. (Shanghai, China).

### 4.4. Bioinformatics and Statistical Analysis

Bioinformatics processing of the raw sequencing reads was primarily conducted using the QIIME 2 (v2024.5) platform [54]. The cutadapt plugin was used to trim primers and adapter sequences [55]. The DADA2 algorithm was applied for quality filtering, denoising, paired-end merging, and chimera removal, generating high-resolution Amplicon Sequence Variants (ASVs, equivalent to Operational Taxonomic Units clustered at 97% similarity) [56]. Taxonomic annotation was performed using a Naive Bayes classifier, aligning bacterial and fungal sequences against the SILVA (Release 138) [57] and UNITE (Release 9.0) [58] reference databases, respectively.

Statistical analyses were conducted using R software v4.3.3 (R Core Team, Vienna, Austria) [59] and SPSS 26.0 (IBM Corp., Armonk, NY, USA). To evaluate the overall rhizosphere ecological effects throughout the entire plant growth season and to mitigate the interference of short-term temporal fluctuations, the data from the eight temporal sampling points (April to October) for each biological replicate were averaged prior to the downstream statistical analyses (e.g., one-way ANOVA) and bioinformatics evaluations. Alpha diversity (Chao1, Shannon, and Simpson indices) and Beta diversity (PCoA based on Bray–Curtis distance) were used to evaluate the successional characteristics of community structures. Linear Discriminant Analysis Effect Size (LEfSe) with a logarithmic LDA score threshold of 4.0 was employed to identify core differential microbial biomarkers under various invasion modes. Spearman rank correlation coefficients were used to construct a heatmap associating cross-kingdom core microbial functional guilds with soil physicochemical properties/enzyme activities. The correlation coefficient thresholds were determined via Random Matrix Theory to reveal the intrinsic driving mechanisms between soil nutrient mobilization and microecological restructuring [60,61]. The threshold for statistical significance was set at *p* < 0.05.

### 4.5. Use of Generative Artificial Intelligence

In accordance with the journal’s guidelines, the authors disclose that generative artificial intelligence (GenAI) was used in the preparation of the conceptual model (Figure 5). The AI tool Gemini (Google LLC, Mountain View, CA, USA) was employed to generate the non-scientific aesthetic elements, including the background sky, soil textures, and the initial stylistic rendering of the plant figures. However, all critical biological and scientific entities—specifically the morphological structures of *Solanum rostratum* and *Cenchrus pauciflorus*, the architecture of the root systems, and the microbial nodes—along with the mechanistic pathways and final conceptual composition, were manually designed, verified, and integrated by the authors using Adobe Illustrator 2022 (Adobe Inc., San Jose, CA, USA) to ensure scientific accuracy. GenAI was not used for study design, data collection, analysis, or interpretation. The authors take full responsibility for the scientific integrity, originality, and accuracy of all content in this manuscript.

## 5. Conclusions

This study reveals the underlying mechanisms by which microecological restructuring drives the asymmetric rhizosphere microecological responses of co-invading plants in oligotrophic sandy habitats. Data confirm that under intense resource competition and shared pathogen stress, environmental filtering compels the overall bacterial community structures of both competitors to undergo significant convergent succession. Conversely, owing to their tighter micro-niche associations with the host, fungal communities exhibit asynchronous cross-kingdom assembly responses, retaining partial host specificity.

Against the backdrop of overall community convergence, the establishment of competitive dominance by the dominant species (*Solanum rostratum*) stems not from a comprehensive remodeling of the basal microecological architecture but rather relies on the precise, targeted recruitment of cross-kingdom phosphate-solubilizing functional consortia (*Bacillus* and *Penicillium*). Coupled with the significant upregulation of rhizosphere sucrase activity, this targeted microecological assembly pattern suggests that the host plant likely employs a proactive “carbon investment” strategy through increased root exudation to secure efficient enzymatic phosphorus mineralization. Concurrently, the highly significant negative correlation between core growth-promoting consortia and soil’s available phosphorus content implies that mobilized phosphorus undergoes rapid turnover in extremely barren habitats; specifically, it is rapidly absorbed and assimilated by the potent “nutrient sink” of the competitively dominant plant.

In stark contrast, the comprehensive suppression of the subordinate species (*Cenchrus pauciflorus*) during competition is directly accompanied by the significant loss of its native core rhizosphere stress-resistant taxa (*Rubrobacter*) and the substantial decline of its basal phosphatase supply system. Consequently, we conclude that the competitive interplay between alien invasive species in extreme habitats is essentially a rhizosphere microecological interaction process characterized by the synergistic co-evolution of “overall community structural homogenization” and the “targeted restructuring of specific cross-kingdom functional modules.”

Despite these ecological insights, we acknowledge that our current inferences regarding active carbon allocation and microbial nutrient mobilization are primarily based on extracellular enzyme activity phenotypes and amplicon sequencing. Future studies should prioritize the integration of spatial metabolomics and stable isotope probing (e.g., ^13^C-DNA SIP) to provide direct, causal evidence for root-microbiome carbon–phosphorus material fluxes. Additionally, validating the causal roles of these recruited cross-kingdom consortia through synthetic community (SynCom) inoculation experiments will be crucial. Ultimately, these future endeavors will facilitate the development of precision ecological management strategies, such as targeting specific microecological networks, to effectively control multi-species synergistic invasions in fragile ecosystems.

## Data Availability

The raw 16S rRNA and ITS amplicon sequencing data for *Solanum rostratum* and *Cenchrus pauciflorus* rhizosphere soil presented in this study are openly available in the NCBI Sequence Read Archive (SRA) database. The data can be accessed under the BioProject accession number PRJNA1466033.

## Supplementary Materials

The following supporting information can be downloaded at: https://www.mdpi.com/article/10.3390/plants15121837/s1. Figure S1: Taxonomic composition of rhizosphere bacterial and fungal communities at the phylum level for *Solanum rostratum* and *Cenchrus pauciflorus* under mono- and co-invasion; Table S1: Physicochemical properties and enzyme activities of bulk and rhizosphere soils across different invasion habitats.
