# Supplementary material for: Targeted Recruitment of Cross-Kingdom Phosphate-Solubilizing Microbes Drives Asymmetric Rhizosphere Responses Between Solanum rostratum and Cenchrus pauciflorus Benth. in Sandy Habitats
Source: Plants (Basel). 2026 Jun 14;15(12):1837. doi: 10.3390/plants15121837 (PMC13306262; doi:10.3390/plants15121837)
Supplement: Supplementary file 1 [file plants-15-01837-s001.zip › Figure S1.pdf]

plants

MDPI

Supplementary Materials

Title: Targeted Recruitment of Cross-Kingdom Phosphate-Solubilizing Microbes Drives Asymmetric Rhizosphere Responses between *Solanum rostratum* and *Cenchrus pauciflorus* Benth. in Sandy Habitats

Authors: Song Yang, Zhen Niu, Yilang Miao, Yujie Chen, Guangchao Lyu, Wenjing Ma, Yang Wang, Linyou Lyu, Xun Tian *

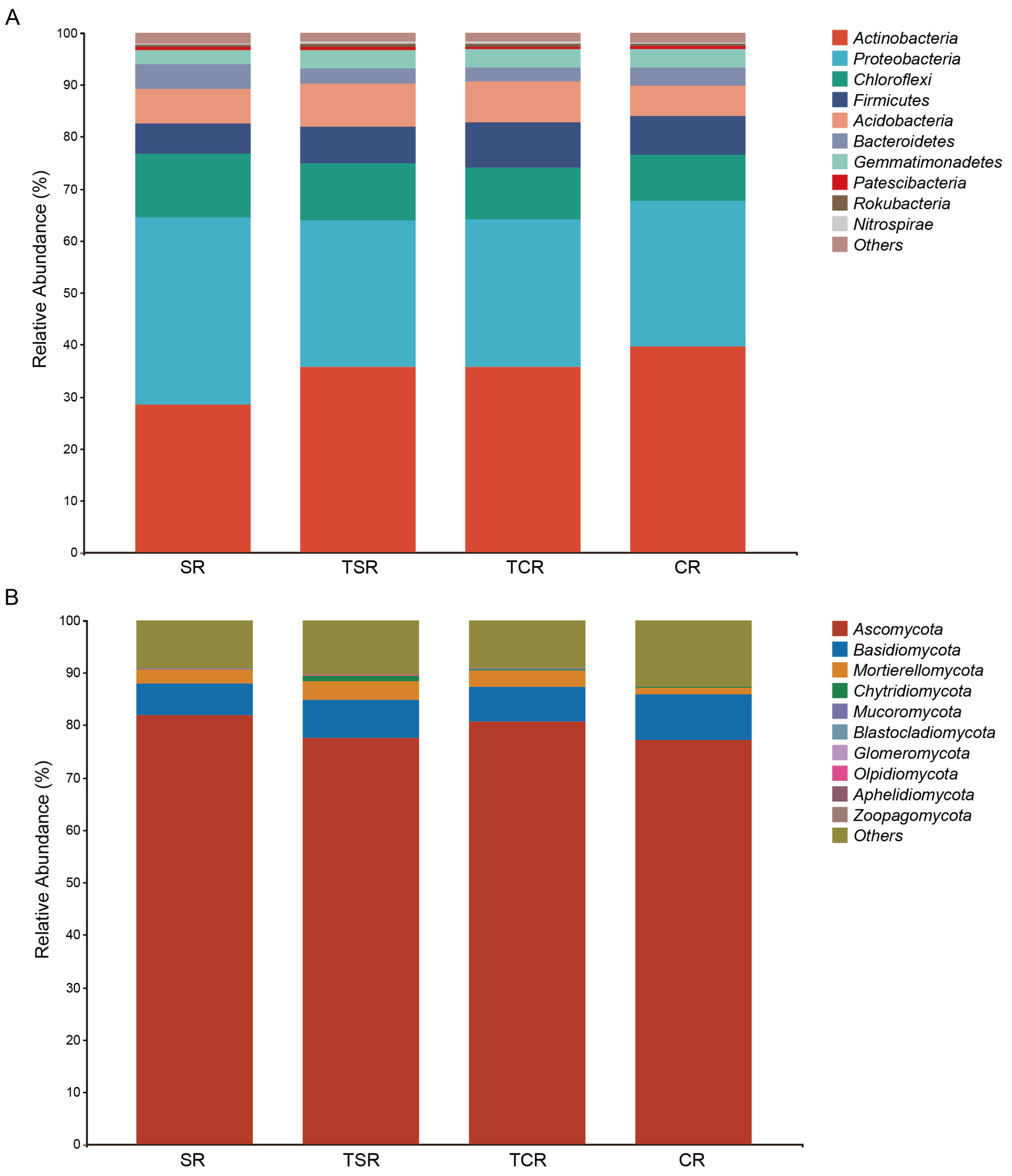

Figure S1. Taxonomic composition of rhizosphere microbial communities at the phylum level. Stacked bar charts illustrate the relative abundances of the top 10 most abundant bacterial (A) and fungal (B) phyla across the different invasion treatments. Phyla outside the top 10 in relative abundance are aggregated into the 'Others' category. Abbreviations: SR, *Solanum rostratum* mono-invasion; TSR, *S. rostratum* co-invasion; CR, *Cenchrus pauciflorus* mono-invasion; TCR, *C. pauciflorus* co-invasion.
